# Factors Influencing Implementation of eHealth Technologies to Support Informal Dementia Care: An Umbrella Review

**DOI:** 10.1093/geroni/igab046.3393

**Published:** 2021-12-17

**Authors:** Sofia Bastoni, Christian Wrede, Marcia C da SIlva, Robbert Sanderman, Andrea Gaggioli, Annemarie Braakman-Jansen, Lisette van Gemert-Pijnen

**Affiliations:** 1 University of Twente, Enschede, Overijssel, Netherlands; 2 University of Twente, Dept. of Psychology, Health & Technology, Enschede, Overijssel, Netherlands; 3 University of Twente, Esnchede, Overijssel, Netherlands; 4 Universitá Cattolica del Sacro Cuore, Universitá Cattolica del Sacro Cuore, Lombardia, Italy

## Abstract

The increase of People with Dementia (PwD) living at home underscores the need for innovative eHealth technologies that support both patients and informal caregivers (IC). Sustainable implementation of eHealth technologies within this target group can, however, be difficult. Our study aims at providing an overview of (1) technologies employed in the context of informal dementia care (IDC) and (2) factors influencing the implementation of these technologies. Five databases were searched for (systematic) reviews. 21 reviews were included. A combination of deductive and inductive thematic analysis was performed, using the NASSS Framework to organize the findings. We identified technologies used “by IC”, “by PwD” and “with PwD”. Most represented technologies included: (i) devices for in-home monitoring (ii) technologies for supporting memory, orientation, and day structure, and (iii) communication technologies. Most factors influencing implementation related to the condition of dementia, characteristics of the technology, the expected/perceived value by users, and the characteristics of the IC. Considerably less has been reported on factors related to the implementing organization, the technology supplier, the wider institutional and sociocultural context of policy and regulations, and the adaptation of technology over time. Our study 1) created a comprehensive overview of eHealth technologies employed in the context of IDC and contributes to a better understanding of factors influencing their implementation, and 2) uncovered a knowledge gap regarding success factors for implementation related to the wider context. Although future research is needed, these findings can help researchers improving the development and implementation of eHealth technologies to support IDC.

